# A possible crosstalk between cGMP pathway and Activin receptor-like kinase 7 (Alk7) in adipocytes

**DOI:** 10.1186/2050-6511-14-S1-P4

**Published:** 2013-08-29

**Authors:** Aileen Balkow, Johanna Jagow, Ana Kilic, Alexander Pfeifer

**Affiliations:** 1 Institute of Pharmacology and Toxicology, University of Bonn, 53105 Bonn, Germany

## Background

In last decades, obesity became a major health issue worldwide. Processes as well as molecules involved in fat mass regulation remain unresolved up to now. Brown adipose tissue (BAT) is an interesting target to fight obesity, especially since brown adipose tissue in adult humans was recently “rediscovered”. In addition, the occurrence of “brown-like” adipocytes within white adipose tissue (WAT) has also sparked an interest due to potential anti-obese effect. Transforming growth factor-β (TGF-β) superfamily consists of structurally related but functionally diverse cytokines involved in regulation of adipogenesis. Activin receptor-like kinase-7 (Alk7), a member of the TGF-ß family, is highly expressed in adipose tissue. However, its role in adipogenesis, thermogenesis and adipocyte function is not well known.

## Results

Here, we analyzed regulation of both brown and white adipocytes focusing on Alk7 pathways. Levels of Alk7 mRNA are almost negligible at the beginning of differentiation protocol. Interestingly, expression of Alk7 increases with differentiation of brown and white adipocytes and reaches highest expression levels close to the end of differentiation protocol. Chronic treatment with 8-pCPT-cGMP (200 µM) increases expression of Alk7 in both brown and white adipocytes. Expression of Alk7 mRNA by cGMP is regulated *via* activation of PKGI, since PKGI KO adipocytes show reduced levels of Alk7. Moreover, our preliminary data suggest involvement of PKGI in Alk7 promoter activation.

Presently we are investigating effects of Alk7 activation in the presence and absence of cGMP onto adipocyte differentiation and BAT function.

## Conclusion

Taken together, our data show that cGMP via PKGI modulates expression of Alk7 in both white and brown adipocytes, thus pointing towards Alk7 as a new PKGI target.

Considering expression pattern of Alk7, this interaction could prove beneficial for developing new pharmacological treatments to fight obesity.

